# Co‐Culture Systems to Study Epithelial‐Immune Interactions During SARS‐CoV‐2 Infection

**DOI:** 10.1002/cpz1.70365

**Published:** 2026-04-08

**Authors:** Scott H. Randell, Katherine C. Barnett

**Affiliations:** ^1^ Marsico Lung Institute University of North Carolina at Chapel Hill, Chapel Hill North Carolina; ^2^ Department of Microbiology and Immunology University of Michigan Medical School Ann Arbor Michigan

**Keywords:** human airway epithelium, inflammasome, interleukin‐1β, interleukin‐6, peripheral blood mononuclear cells, SARS‐CoV‐2

## Abstract

Robust inflammatory responses to viral infection are mediated by immune cell populations, including monocytes and dendritic cells. However, severe acute respiratory syndrome coronavirus 2 (SARS‐CoV‐2) does not replicate efficiently in these cell types and instead preferentially infects epithelial cell subsets in the airway. Because of this, stimulation of inflammatory cytokine responses from immune cell populations during SARS‐CoV‐2 infection depends not only on interactions with viral particles but also interactions with infected epithelial cells. In this article, we describe two co‐culture systems to study inflammatory cytokine responses generated by epithelial‐immune interaction during SARS‐CoV‐2 infection in vitro. Basic Protocol 1 describes how to set up a partially primary co‐culture system consisting of SARS‐CoV‐2‐infected Vero‐E6 cells and primary human peripheral blood mononuclear cells (PBMCs) to observe release of the inflammasome‐regulated cytokine interleukin‐1β (IL‐1β). Basic Protocol 2 details a primary, human co‐culture system that consists of SARS‐CoV‐2‐infected primary human airway epithelia (HAE) grown at an air–liquid interface (ALI) and primary human PBMCs, and how to observe IL‐1β and IL‐6 crosstalk between these cell populations during infection. In these Basic Protocols, we include a description of the use of inhibitors in these systems to perturb cytokine responses. We also provide Support Protocols for the culture of HAE and Vero‐E6 and for the isolation, storage, and preparation of PBMCs prior to use in these systems. © 2026 The Author(s). Current Protocols published by Wiley Periodicals LLC.

**Basic Protocol 1**: SARS‐CoV‐2 infection in a Vero‐E6+PBMC co‐culture system

**Support Protocol 1**: Vero‐E6 culture and passaging

**Support Protocol 2**: Isolation and cryopreservation of PBMCs for use in co‐culture

**Basic Protocol 2**: SARS‐CoV‐2 infection in a primary HAE+PBMC co‐culture system

**Support Protocol 3**: Establishment and maturation of HAE grown at an ALI

## INTRODUCTION

Understanding the mechanisms by which SARS‐CoV‐2 stimulates inflammation is critical to our understanding of coronavirus disease 2019 (COVID‐19), which remains a significant public health burden (Sherman et al., 2025). Timely inflammatory responses are required for the development of protective adaptive immunity. However, excessive inflammation may exacerbate and contribute to certain viral disease states, such as the acute respiratory distress syndrome (ARDS) associated with COVID‐19 fatalities and the development of post‐acute sequalae of COVID‐19 (PASC) or long COVID‐19 (Bartsch et al., 2025; Merad et al., 2022; Narota et al., 2022). Thus, a thorough understanding of how inflammation arises during SARS‐CoV‐2 infection is necessary to understand COVID‐19 progression.

Robust inflammatory responses are mediated most frequently by myeloid cell types, including monocytes and dendritic cells, but in the human airway, SARS‐CoV‐2 most efficiently infects epithelial cell types (Hou et al., 2020), which express high levels of angiotensin‐converting enzyme 2 (ACE2) and transmembrane protease, serine 2 (TMPRSS2) that facilitate SARS‐CoV‐2 entry into cells (Hoffmann et al., 2020; J. Lan et al., 2020). By contrast, SARS‐CoV‐2 does not replicate efficiently in myeloid cell types due to a lack of high ACE2 expression (Rockx et al., 2020; Zhao et al., 2020). Despite this, SARS‐CoV‐2 infection causes robust inflammatory responses (McGonagle et al., 2020; Mulchandani et al., 2021), including production of the inflammatory cytokines IL‐1β and IL‐6 that are implicated in severe COVID‐19 and COVID‐19 fatalities.

Because SARS‐CoV‐2 does not directly infect myeloid cells, studying the specific cellular and molecular mechanisms that govern inflammatory responses to SARS‐CoV‐2 is challenging. Our work and the work of others have shown that myeloid cells are stimulated during SARS‐CoV‐2 infection through interaction with infected epithelial cells (Barnett et al., 2023; Chua et al., 2020). While in vivo infection models provide an avenue for investigation of these cell–cell interactions during infection, animal models are expensive, time‐consuming, and cannot fully replicate the biology of humans. This is especially important for SARS‐CoV‐2, which preferentially binds human ACE2 (Wrobel, 2023; Zhou et al., 2020). Because of this, animal models of COVID‐19 require genetic manipulation of the virus and/or the host to study infection in vivo, limiting their fidelity to human COVID‐19 patients. Moreover, specific experimental designs, such as drug screenings and detailed mechanistic studies, are more suitable for cell culture systems than animal models. Thus, in vitro models of epithelial‐immune cell interactions in SARS‐CoV‐2 infection are a valuable tool for researchers interested in studying human‐specific responses, investigating the molecular mechanisms of inflammation, and conducting high‐throughput experiments.

Here, we outline established methods for two epithelial‐immune co‐culture systems in the study of inflammatory responses to SARS‐CoV‐2 infection (Barnett et al., 2023). The first method outlined in Basic Protocol 1 is a rapid, cost‐effective, partially primary co‐culture system that utilizes the Vero‐E6 cell line, one of the most widely used epithelial cell lines in SARS‐CoV‐2 research (Wang et al., 2021), and primary human PBMCS, which contain multiple myeloid cell populations that drive inflammatory responses in COVID‐19 (Oelen et al., 2022). To accompany this method, we describe how to culture and plate the Vero‐E6 line in Support Protocol 1, and we describe how to isolate, store, and culture PBMCs in Support Protocol 2. This system is effective specifically for studying how primary human immune cells respond to virally infected cells and can be used to interrogate the inflammasome‐mediated IL‐1β response to SARS‐CoV‐2 infection (Barnett et al., 2023). However, because Vero‐E6 cells are an immortalized simian epithelial line, this partially primary system has limited value in the study of how epithelial cells respond to immune cells during infection. For this reason, the second method we describe in Basic Protocol 2 is a fully primary human epithelial‐immune co‐culture system that consists of primary human airway epithelia (HAE) and primary human PBMCs. Primary HAE are primary human bronchial epithelial (HBE) cells grown on permeable cell culture supports at an air–liquid interface that recapitulate the pseudostratified mucociliary epithelial morphology of the human large airways, and we describe their 28‐day culture process in Support Protocol 3. This fully primary system enables the study not only of how human immune cells respond to SARS‐CoV‐2 infected epithelial cells but also how the epithelial cells respond in turn to stimuli from the immune compartment, though it is more time‐consuming and resource intensive. With this system, we can observe not only release of the inflammasome‐mediated cytokine IL‐1β from human immune cells but also the IL‐1‐dependent cytokine IL‐6, which is released from the epithelial cells in addition to immune cells (Barnett et al., 2023). Together, these systems can be used to interrogate many different questions related to how human immune cells interact with SARS‐CoV‐2‐infected epithelial cells and how cell–cell interactions drive inflammatory responses during infection. The protocols described here have been consistent and worked well for our research, but we also provide guidance for optimization and troubleshooting when it may be warranted.

## STRATEGIC PLANNING

**Figure 1:**
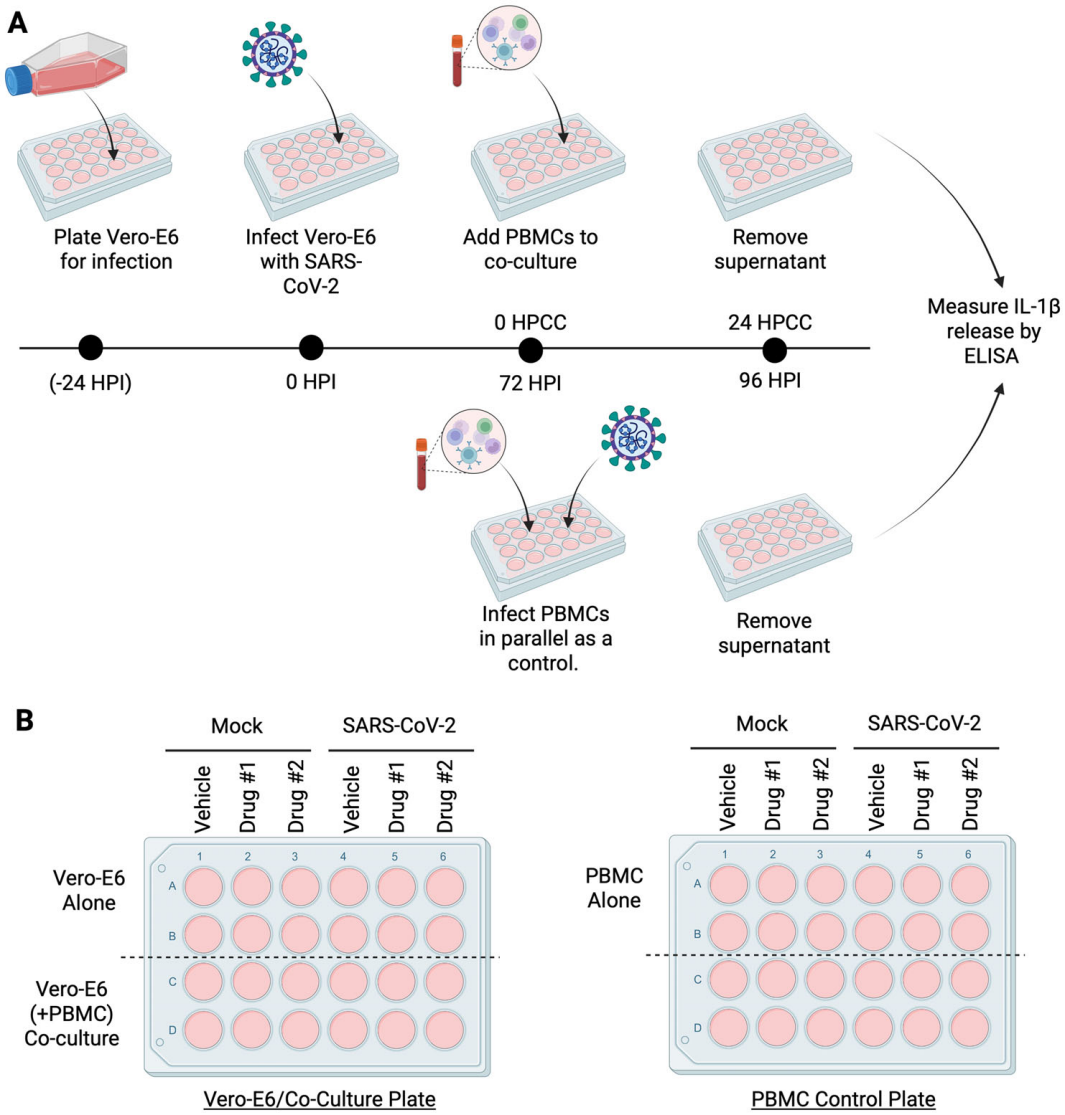
Overview of partially primary co‐culture system in Basic Protocol 1. (**A**) Experiment timeline: 24 hr before the start of the experiment, Vero‐E6 cells are plated, and these cells are infected with SARS‐CoV‐2 the following day. At 72 hr post‐infection (HPI) of the Vero‐E6 cells, which begin to show CPE at this timepoint, PBMCs are added to the specified wells for co‐culture, and in parallel, PBMCs are infected with SARS‐CoV‐2 as a control. At 24 hr post‐coculture (HPCC) (96 hr after the initial infection), cell culture supernatants are collected and assayed by ELISA for IL‐1β release. (**B**) Plate set up: One plate of Vero‐E6 (left) will be infected or mock‐infected for Vero‐E6 alone controls and the co‐culture conditions. A second plate (right) will be used for PBMC alone controls. Duplicates are suggested for technical replicates and drugs can be tested as shown. Created in BioRender. Barnett, K. (2006) https://BioRender.com/9k231ew.

**Figure 2:**
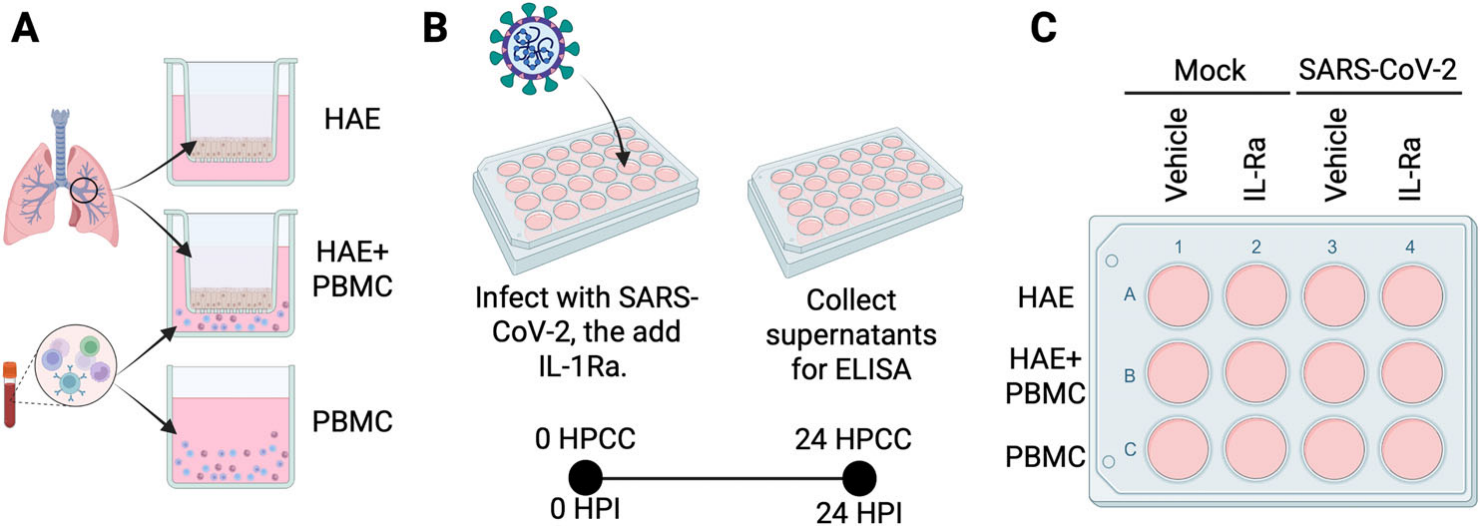
Overview of primary human co‐culture system in Basic Protocol 2. (**A**) Schematic: Primary HAE and PBMCs are cultured alone or in co‐culture in a 12‐well plate. HAE are grown on Transwell inserts, while PBMCs are grown in suspension culture. For the co‐culture condition, PBMCs are placed the basolateral medium below the HAE grown at an air–liquid interface. (**B**) Experiment timeline: 24 hr before the start of the experiment, wash the HAE ALI surface to remove accumulated mucus. The next day, infect HAE/PBMC with SARS‐CoV‐2 and set up plates with HAE‐ or PBMC‐only and HAE+PBMC co‐culture conditions. IL‐1Ra is added at the end of the infection period as described in the protocol. After 24 hr, collect supernatants for analysis by ELISA for cytokine responses. (**C**) Plate set up: In a 12‐well plate, culture HAE, PBMC, or HAE+PBMC as shown in (**A**). Mock‐infect or infect each group of cell types with or without the IL‐1Ra. Created in BioRender. Barnett, K. (2026) https://BioRender.com/e3r5s5a.

Because these co‐culture systems require viral infections and multiple cell types that use different methods of culture or isolation, researchers must take special care when planning the timing and execution of these experiments. To aid in understanding the specific needs of these experiments, Figures 1 and 2 provide overviews of the timelines of the experiments outlined in Basic Protocols 1 and 2. To aid in understanding the specific needs of each cell type involved, Support Protocol 1 outlines the isolation and storage of PBMCs prior to thawing these cells for the co‐culture experiments in Basic Protocols 1 and 2, and Support Protocols 2 and 3 outline the culture methods for immortalized Vero‐E6 cells and primary HAE.

*CAUTION*: SARS‐CoV‐2 is handled at Biosafety Level 2 (BSL‐2) or higher following updated guidance from the United States of America Centers for Disease Control that enabled handling of SARS‐CoV‐2 at BSL‐2 instead of BSL‐3 at the discretion of your institution. Follow all appropriate guidelines and regulations for the use and handling of pathogenic microorganisms, as dictated by your institution.

*CAUTION*: All human cells are handled at BSL‐2. Follow all appropriate guidelines and regulations for the use and handling of human cells.

*CAUTION*: All human blood products are handled at BSL‐2. Follow all appropriate guidelines and regulations for the use and handling of human blood products.

*NOTE*: All materials, solutions, and equipment must be sterile, all cell culture must be performed in a biosafety cabinet (BSC), and proper aseptic techniques must be used accordingly.

*NOTE*: All protocols involving animals must be reviewed and approved by the appropriate Animal Care and Use Committee and must follow regulations for the care and use of laboratory animals. Appropriate informed consent is necessary for obtaining and use of human study material.

## Basic Protocol 1 SARS‐CoV‐2 INFECTION IN A Vero‐E6+PBMC CO‐CULTURE SYSTEM

By co‐culturing infected Vero‐E6 cells with primary human PBMCs, inflammatory responses to SARS‐CoV‐2‐infected cells can be observed and measured to understand how infected cells stimulate inflammatory responses from immune cells during infection. To accomplish this, PBMCs are added to infected Vero‐E6 cultures when cytopathic effects (CPE) are first observed, at 72 hr post‐infection (HPI) in our hands, and then cultured for an additional 24 hr. Following this culture period, cytokine release driven by cell–cell interaction, including the inflammasome‐regulated cytokine IL‐1β, can be detected by ELISA, which can be modified by the addition of inhibitor compounds, if desired. Figure 1A provides a graphic overview of the protocol timeline. Figure 1B provides a sample plate setup for this experiment.

### Materials

Vero‐E6 cells, early passage (ATCC, cat. no. CRL‐1586) (see Support Protocol 1)
Phosphate‐buffered saline (PBS) (e.g., Gibco, cat. no. 10010023)
0.25% trypsin‐EDTA (e.g., Gibco, cat. no. 25200056)
Complete minimal essential medium (MEM), supplemented with 10% fetal bovine serum (FBS) (see recipe)
SARS‐CoV‐2 virus stocks, e.g., WA‐1 variant (BEI, cat. no. NR‐52281)
Virus diluent, complete MEM supplemented with 2% FBS (see recipe)
Fresh or frozen PBMCs (see Support Protocol 2)
Drug compounds, e.g., VX‐765 resuspended at 20 mg/ml (see recipe), if desired
Solvents for drug compounds, e.g., dimethyl sulfoxide (DMSO) (D2650‐100ML)
Human IL‐1β ELISA Set II (e.g., BD Biosciences, cat. no. 557953)

75‐cm^2^ tissue culture (T75) flask
Type II class A2 biosafety cabinet
Incubator, 37°C, 5% CO_2_
Inverted microscope
15‐ml conical tube
Cell counter or hematocytometer
24‐well tissue culture treated plate
Tabletop centrifuge
Filtered pipet tips
Water bath, 37°C
Microcentrifuge tubes
Microcentrifuge
Plate reader capable of measuring absorbance

#### Plate Vero‐E6 cells for infection

1 Take a T75 flask of Vero‐E6 cells that have reached >90% confluence.
2 Working in a biosafety cabinet, remove culture medium, rinse with 10 ml PBS, remove PBS, and add 2.5 ml of 0.25% trypsin‐EDTA to the culture surface. Place flask in an incubator at 37°C and 5% CO_2_ for 5 min to dissociate cells.
3 Confirm cells have dissociated from the culture surface using an inverted microscope.
4 Working in a biosafety cabinet, add 7.5 ml complete MEM with 10% FBS to neutralize the trypsin, resuspend the cells, place in a 15‐ml conical tube, and take an aliquot for counting cells.
5 Count the cells using a cell counter or hematocytometer. Determine the number of cells needed to plate a 24‐well tissue culture plate at 100,000 cells/well, according to the formula below. Add an additional well to these calculations to account for pipetting error.
$$\frac{\#\frac{desired\;cells}{well}\;\times \;\#\;wells}{\#\frac{cultured\;cells}{ml}}=\;volume\;of\;cultured\;cells\;needed.$$
  These cells will be >90% confluent after plating and incubating overnight for the infection step that follows below.
6 Working in a biosafety cabinet, place the needed amount of cell suspension in a 15‐ml conical tube. Pellet the cells on a tabletop centrifuge for 5 min at 400 × *g*, room temperature.
7 Working in a biosafety cabinet, remove the supernatant and resuspend the cell pellet at 0.5 ml per well (e.g., 12.5 ml if calculating for 25 wells) in complete MEM. After resuspension, place 0.5 ml in each well of a 24‐well tissue culture plate.
8 Shake the plate back‐and‐forth and then side‐to‐side to evenly distribute the cells in plate.
9 Place cells in a tissue culture incubator at 37°C and 5% CO_2_ to adhere and grow overnight.

#### Infect Vero‐E6 cells with SARS‐CoV‐2

10 Working in a biosafety cabinet in a space designated for SARS‐CoV‐2 infection at your institution, prepare the viral inoculum by diluting SARS‐CoV‐2 stocks in virus diluent in a 15‐ml conical tube. Use filter tips when pipetting viral stocks and infected culture medium. Gently pipette up and down to mix.
  For these experiments, use a multiplicity of infection (MOI) of 0.5 and deliver the inoculum to each well at a volume of 150 µl (0.15 ml). Plan to infect half the wells in the plate and mock‐infect the other half. See sample plate setup in Figure 1B. Use the formulas below with provided sample calculations and include an extra well to account for pipetting error in these calculations:
  a. Volume of viral stock needed for infection:
$$\begin{array}{ccc} & & \frac{\#cells\;per\;well\;\times \;number\;of\;wells\;\times \;MOI\;\left(\frac{PFU}{cell}\right)}{viral\;stock\;titer\;PFU/ml}=\\ & & viral\;stock\;volume\;needed \end{array}$$
  b. Volume of virus diluent needed:
$$\begin{array}{ccc} & & \left(\#wells\times inoculum\;volume\;per\;well\right)-viral\;stock\;volume=\\ & & diluent\;volume\;needed \end{array}$$
  c. Sample calculation for viral stock volume:
$$\frac{100,000\frac{cells}{well}\;\times \;13\;wells\;\times \;0.5\frac{PFU}{cell}}{10^{7}\frac{PFU}{ml}}=0.065\;ml$$
  d. Sample calculation for virus diluent:
$$\left(13\;wells\times 0.15\;ml\right)-0.065\;ml=\;1.885\;ml$$
  e. With the sample calculations shown above, virus inoculum would be prepared by placing 0.065 ml (65 µl) SARS‐CoV‐2 virus stock into 1.885 ml virus diluent.
11 Once the viral inoculum is prepared, take the plate of Vero‐E6 cells prepared in the above section.
12 Working in a biosafety cabinet, remove the culture medium from each well and then add 0.5 ml PBS to each well to rinse. For wells to be mock‐infected, remove the PBS from the well and then add 0.15 ml virus diluent. For wells to be infected, removed the PBS from the well and then add 0.15 ml viral inoculum.
  The low volume in these wells is to maximize efficiency to virus uptake by cells.
  Make sure to not cross‐contaminate wells by changing pipette tips between conditions.
13 Gently shake the plate front‐and‐back and then side‐to‐side to distribute the viral inoculum evenly. Place plate in a tissue culture incubator at 37°C and 5% CO_2_.
14 Over a 1‐hr infection period, shake the plate front‐and‐back and then side‐to‐side every 15 min to agitate the viral inoculum.
15 After the 1‐hr infection period, place plate in the biosafety cabinet. Remove virus diluent from mock‐infected wells, add 0.5 ml PBS to rinse the well, remove PBS, and replace with 0.5 ml virus diluent. Then, remove viral inoculum from infected wells, add 0.5 ml PBS to rinse the well, remove PBS, and replace with 0.5 ml virus diluent.
16 Place plate back in the tissue culture incubator at 37°C and 5% CO_2_. Incubate until the co‐culture step described in the section below.

#### Co‐culture set up

17 Monitor the infected Vero‐E6 for CPE daily post‐infection. When significant CPE begins to develop, begin the co‐culture protocol described below. In our hands, this is 72 HPI.
18 Working in a biosafety cabinet, prepare PBMCs for co‐culture and control infections.
  a. If working with fresh PBMCs, count the cells and resuspend at 1 × 10^6^ cells/ml in virus diluent.
  b. If working with cryopreserved PBMCs, thaw rapidly in a 37°C water bath, resuspend in 10 ml warmed complete MEM in a 15‐ml conical tube, count the cells, pellet cells to remove cryoprotectant in a tabletop centrifuge for 5 min at 400 × *g*, room temperature, remove supernatant, resuspend pellet at 1 × 10^6^ cells/ml in warm virus diluent.
19 After resuspending PBMCs, remove the infected Vero‐E6 plate from the incubator.
20 Working in a biosafety cabinet, set up the co‐culture conditions. Add 100 µl of the PBMC suspension to co‐culture wells. This includes mock‐infected and infected wells of Vero‐E6 designated for co‐culture (see Fig. 1B). Change pipet tips between conditions. PBMCs will be added at 1 × 10^5^ cells/well, or a 1:1 ratio with cultured Vero‐E6 cells.
21 Add 100 µl virus diluent to Vero‐E6‐only conditions to control for the change in culture volume in the co‐culture wells.
  If desired, drugs can be spiked into culture wells at this point to test how different compounds alter cell–cell interactions during the co‐culture phase. If drugs are added, vehicle‐alone controls should be used as well (see Fig. 1B), meaning an equal amount of the drug solvent should be added to vehicle wells to control for changes in well volume and medium composition. For instance, if adding the caspase‐1 inhibitor VX‐765, which will block IL‐1β release (Wannamaker et al., 2007), add 1 µl of 1000× stock in DMSO to treatment wells and 1 µl DMSO alone to vehicle wells.
22 Return plate containing Vero‐E6 conditions and co‐culture conditions to the tissue culture incubator at 37°C and 5% CO_2_ following set up.

#### PBMC control infections for co‐culture

23 In parallel, perform control infections in PBMCs. Working in a biosafety cabinet, set up a plate of PBMCs by adding 100 µl of PBMC suspension to each well, using the diagram in Figure 1B as a guide, so that each well contains 1 × 10^5^ PBMCs.
24 Calculate the amount of virus stock needed according to the instructions in step 10a. Because the PBMCs are already in virus diluent at a volume of 100 µl per well, plan to add an additional 50 µl of viral inoculum to each well and calculate the amount of virus diluent needed to dilute the stock volume calculated in the above step using the formula in step 10b.
25 Dilute the viral stock in virus diluent according to your calculations.
26 Add 50 µl of viral inoculum to each PBMC well designated for infection. Add 50 µl of virus diluent to each PBMC well designated for mock‐infection. Change pipet tips between each well to prevent cross‐contamination.
27 Place the plate in the tissue culture incubator at 37°C and 5% CO_2_ for a 1‐hr infection period. Shake the plate every 15 min.
28 Following infection, working in a biosafety cabinet remove viral inoculum.
  a. Place the cell suspension from each well in microcentrifuge tubes, pellet cells in a microcentrifuge for 5 min at 500 × *g*, room temperature, and remove supernatant.
  b. Wash each pellet by gently resuspending in 0.5 ml PBS, pellet the cells again for 5 min at 500 × *g*, room temperature, remove the supernatant, and then resuspend in 0.6 ml virus diluent to match the volume of Vero‐E6‐only and co‐culture conditions.
  c. Place resuspended cells into a fresh, labeled 24‐well plate. If desired, add drugs to wells as described for the Vero‐E6 and co‐culture conditions as described in step 21 above.
29 Place plate in tissue culture incubator at 37°C and 5% CO_2_ following set up.
  To optimize timing, the PBMC infections can be started and then the co‐culture conditions can be set up during the PBMC infection period.
30 Incubate the Vero‐E6 alone, co‐culture, and PBMC alone plates for 24 hr.

#### Collect culture supernatants for ELISA

31 Working in a biosafety cabinet, collect supernatants from each well by removing medium/cells from each well and placing in a microcentrifuge tube.
32 Place each tube in a microcentrifuge and pellet for 5 min at 500 × *g*, room temperature, to remove cells and debris.
33 Place supernatants from these tubes into fresh microcentrifuge tubes for further analysis. These samples can be frozen at −20°C until proceeding to step 34.
34 Measure IL‐1β release in supernatants using the Human IL‐1β ELISA Set II according to the manufacturer's instructions. Use a plate reader to measure IL‐1β, as directed by the kit instructions.
  If successful, only conditions with both PBMCs and Vero‐E6 present will show IL‐1β release upon SARS‐CoV‐2 infection, showing how release of inflammasome‐regulated cytokines is stimulated by cell–cell interaction during infection. This response will be blocked if an inhibitor of the inflammasome, e.g., VX‐765, is added to the well during co‐culture. Other compounds and cytokines can be tested in a similar fashion, as desired.
  Because these supernatants contain live SARS‐CoV‐2, the ELISAs must be performed in the biocontainment level appropriate for SARS‐CoV‐2 at your institution.

## Support Protocol 1 Vero‐E6 CELL GROWTH AND PASSAGING

Vero‐E6 cells are an African Green Monkey kidney epithelial cell line that are grown in adherent culture. Here, we detail how to thaw a frozen stock and maintain Vero‐E6 cells in passage. These cells are used in Basic Protocol 1, as described above.

### Materials

Complete MEM, supplemented with 10% FBS (see recipe)
Cryopreserved Vero‐E6 cells, early passage (ATCC, cat. no. CRL‐1586)
PBS (e.g., Gibco, cat. no. 10010023)
0.25% trypsin‐EDTA (e.g., Gibco, cat. no. 25200056)

Water bath, 37°C
Type II class A2 biosafety cabinet
15‐ml conical tube
Centrifuge
75‐cm^2^ tissue culture (T75) flask
Incubator, 37°C and 5% CO_2_
Inverted microscope

#### Thawing cryopreserved Vero‐E6 cells

1 Warm complete MEM with 10% FBS in a water bath or bead bath set to 37°C.
2 Take vial of cryopreserved Vero‐E6 cells from liquid nitrogen storage and rapidly thaw in a water bath at 37°C. Remove from the water bath just before the frozen stock fully melts.
3 Working quickly in a biosafety cabinet, place 9 ml warm complete MEM with 10% FBS in a 15‐ml conical tube and then place the thawed Vero‐E6 cell stock into the medium in the tube.
4 Centrifuge the 15‐ml conical tube to remove cryoprotectant for 5 min at 400 × *g*, room temperature.
5 Following centrifugation and working in a biosafety cabinet, remove the supernatant, resuspend the cell pellet in a fresh 10 ml of complete MEM with 10% FBS, and place in a T75 flask.
6 Distribute the cells evenly on the adherent culture surface of the T75 flask by gently shaking the flask in a cross pattern (up/down and left/right) and place in a tissue culture incubator at 37°C and 5% CO_2_ to grow overnight.
7 The next day assess the cells using an inverted microscope.
  a. If a lot of dead, floating cells are present, remove the old medium, replace with 10 ml of fresh complete MEM with 10% FBS, and return the cells to the incubator.
  b. If cells are >90% confluent, proceed to passage the cells as described below.
  c. Otherwise, return the cells to the incubator, check daily by microscope to assess confluency, and passage as described below when the cells are >90% confluent.

#### Passaging Vero‐E6 cells

8 Working in a biosafety cabinet, remove culture medium from the T75 containing confluent Vero‐E6 cells. Rinse the cell surface with 5 ml PBS to remove any traces of culture medium and then remove the PBS. Following this, place 2.5 ml of 0.25% trypsin‐EDTA on the cell growth surface.
9 Place flask in a tissue culture incubator at 37°C and 5% CO_2_ to support trypsinization for 5 min.
10 Visually inspect the cells using an inverted microscope to confirm detachment from the culture surface. Incubate longer if the cells are not fully detached, inspecting every 1 to 2 min to prevent over trypsinization.
11 Once cells have detached, add 7.5 ml complete MEM with FBS to inactivate the trypsin and gently resuspend cells by pipetting up and down.
12 To maintain Vero‐E6 lines, plan to passage the cells at a 1:10 split ratio. For this, take 1 ml of the 10 ml cell suspension and place it in a 15‐ml conical tube. Centrifuge this tube to pellet cells for 5 min at 400 × *g*, room temperature.
  Different split ratios can be used to expand cells to larger flasks or for use in experiments. Passaging Vero‐E6 at 1:10 ratio will allow for ∼4 days of growth between passages.
13 Remove the supernatant, resuspend cells in 10 ml of fresh complete MEM with 10% FBS, and place this cell suspension in a new T75 flask.
14 Shake flask in a cross pattern (up/down and left/right) and place a tissue culture incubator at 37°C and 5% CO_2_ to grow. Monitor cells regularly with an inverted microscope to assess growth and health. When confluent, cells can be passaged again or plated for an experiment, as described in Basic Protocol 1.

## Support Protocol 2 ISOLATION AND CRYOPRESERVATION OF PBMCS FOR USE IN CO‐CULTURE

PBMCs are easily isolated from whole blood or from leukapheresis packs (leukopaks) obtained during isolation of platelets during leukapheresis, making PBMCs an accessible option for studying primary human immune cells. PBMCs contain several leukocyte populations, including lymphocytes, monocytes, and dendritic cells, which can be used to study the function of these cells in different contexts, including the experiments outlined in Basic Protocols 1 and 2. Here, we will describe how to isolate PBMCs from whole blood or leukopaks and cryopreserve them for use in future experiments. These procedures are described in other Current Protocols articles, e.g., Lauer et al. (2017) and K. Lan et al. (2007), which we encourage the reader to pursue for more details.

### Materials

Human whole blood or leukopaks, obtained from a blood bank or clinic
RPMI 1640 (Gibco, cat. no. 11875093)
Ficoll‐Paque PLUS (Cytiva, cat. no. 17144002)
1× red blood cell (RBC) lysis buffer (Invitrogen, cat. no. 00‐4333‐57)
Hanks’ balanced salt solution (HBSS) (Gibco, cat. no. 14025092)
PBS (e.g., Gibco, cat. no. 10010023)
Freezing medium (see recipe)

Type II class A2 biosafety cabinet
50‐ml syringe
18G needle
50‐ml conical tubes
Centrifuge with adjustable acceleration and deceleration speeds
Sterile wide‐mouth transfer pipette
100‐µm cell strainer
Cell counter or hematocytometer
Inverted microscope
Cryopreservation tubes
Cell freezing container
–80°C freezer
Liquid nitrogen storage (optional)

#### Isolation and freezing of PBMCs

1 Working in a biosafety cabinet, surface decontaminate the whole blood or leukopak container aggressively, remove blood from container, and place in 50‐ml conical tubes.
  a. If blood is in a tube, this can be transferred using a pipette.
  b. If blood is in a sealed plastic bag, carefully remove the blood from the bag by puncturing with an 18G needle fitted on a 50‐ml syringe, draw the blood up through the syringe, and then transfer the blood to the 50‐ml conical tube using a pipette or pouring if the opening is too small to fit a pipette tip.
    Exercise caution when using sharps with human blood products. A sterile, pyrogen‐free needle ensures no endotoxin contamination occurs at this step. Alternatively, endotoxin‐free, sterile scissors may be used to cut open the top of the blood bag. Follow all biosafety guidance for working with human blood products at your institution.
2 If using a leukopak, dilute 1:3 with RPMI 1640. For instance, mix 50 ml blood with 100 ml medium.
  This is not necessary for whole blood.
3 For each 30 ml of blood or diluted leukopak, place 15 ml Ficoll‐Paque PLUS in a 50‐ml conical tube.
4 Gently layer 30 ml of blood or diluted leukopak onto the 15 ml Ficoll‐Paque PLUS in each conical tube.
  Be careful to layer the blood on top of the Ficoll‐Paque PLUS by slowly pipetting the blood down the side of the tube. Do not disrupt the interface between the Ficoll‐Paque PLUS and the blood.
5 Carefully move the tubes to a tabletop centrifuge. Centrifuge samples for 25 min at 800 × *g*, room temperature, with the lowest possible acceleration and deceleration speeds. If possible, turn off the brake entirely.
  Please note that this will take longer than 25 min, as the low acceleration and deceleration speeds will increase the time in the centrifuge. This is necessary to prevent disruption of the buffy coat collected in the following step.
6 During the centrifugation step, place 1× RBC lysis buffer in a water bath at 37°C to warm.
7 Following centrifugation and working in a biosafety cabinet, collect the buffy coat using a sterile wide‐mouth transfer pipette. The buffy coat contains the PBMCs. This is the fuzzy white layer of cells in the tube sitting directly on top of the Ficoll‐Paque PLUS. Transfer to a new 50‐ml conical tube and repeat for all samples. Pooling buffy coats from the same blood sample into the same 50‐ml conical tube.
8 Dilute the collected buffy coat 1:3 with HBSS. For instance, for 10 ml of buffy coat, add 20 ml HBSS. Centrifuge cells for 10 min at 800 × *g*, room temperature.
9 Remove the supernatant and resuspend the cell pellet in 10 ml PBS. Centrifuge cells for 10 min at 800 × *g*, room temperature.
10 Remove the supernatant and resuspend cells with 5 ml of warm 1× RBC lysis buffer at room temperature. Incubate for 5 min.
11 After 5 min, stop the reaction by adding 10 ml PBS. Spin cells for 5 min at 800 × *g*, room temperature. If significant red blood cell contamination is observed in the pellet, repeat steps 10 and 11.
12 Remove the supernatant and resuspend cells in 5 ml PBS. Pass cells through a 100‐µm cell strainer and rinse the strainer with an additional 5 ml PBS.
13 Gently pipette the solution to mix thoroughly and count cells using a hematocytometer and inverted microscope or a cell counter.
14 Pellet cells in a centrifuge for 5 min at 800 × *g*, room temperature. These pelleted cells are ready for use in an experiment, e.g., Basic Protocols 1 and 2, or can be frozen for use later, as described in the following steps.
15 Remove supernatant and resuspend cells in 10 ml freezing medium.
  We freeze PBMCs at ∼5 × 10^7^ cells per cryovial to ensure thawing one vial provides enough cells for the experiments outlined in Basic Protocols 1 and 2, aliquoting 1 ml of cell suspension per vial. The total number of cells isolated will depend on the amount of starting material. Adjust the volume of the freezing medium and the number of cells frozen as needed.
16 Place 1 ml of cell suspension in freezing medium in a labeled cryovial. Repeat for additional cryovials for the entire cell suspension.
17 Place PBMCs in a freezing container and freeze overnight at −80°C. Transfer to liquid nitrogen for long‐term storage. Cells can be revived as needed for use in experiments.

## Basic Protocol 2 SARS‐CoV‐2 INFECTION IN A PRIMARY HAE+PBMC CO‐CULTURE SYSTEM

Here, we detail a fully primary, human co‐culture system to examine intercellular cytokine signaling between the airway epithelial and immune compartments during SARS‐CoV‐2 infection. In this system, primary HAE, which recapitulate the pseudostratified epithelium of the large airway and contain ciliated epithelial cells that SARS‐CoV‐2 preferentially infects (Hou et al., 2020), are cultured with PBMCs, which contain several myeloid cell populations enriched in inflammatory signaling pathways, at the start of infection and then cultured for 24 hr. At 24 HPI/HPCC, supernatants from these cultures can be collected and both IL‐1β and IL‐6 release can be detected by ELISA. IL‐6 release in this system is IL‐1‐dependent and can be antagonized by addition of the IL‐1 receptor antagonist (IL‐1Ra), if desired. Figure 2A shows the setup for mono‐ or co‐culture of PBMCs and HAE, Figure 2B provides an overview of the protocol timeline, and Figure 2C provides a sample plate setup for the experiment.

### Materials

Mature HAE on 12‐mm Transwells (Corning, cat. no. 3460) (see Support Protocol 3)
PBS (e.g., Gibco, cat. no. 10010023)
Low endotoxin UNC (University of North Carolina) ALI medium (see recipe)
Fresh or frozen PBMCs (see Support Protocol 2)
MEM, supplemented with 10% FBS (see recipe)
Virus diluent, complete MEM supplemented with 2% FBS (see recipe)
SARS‐CoV‐2 virus stocks, e.g., WA‐1 variant (BEI, cat. no. NR‐52281)
Human IL‐1Ra, resuspended at 1 µg/µl (see recipe)
IL‐Ra resuspension buffer, PBS with 0.1% (w/v) bovine serum albumin (BSA) (see recipe)
Human IL‐1β ELISA Set II (e.g., BD Biosciences, cat. no. 557953)
ELISA MAX Deluxe Set Human IL‐6 (e.g., Biolegend, cat. no. 430515)

Incubator, 37°C, 5% CO_2_
Water bath, 37°C
Type II class A2 biosafety cabinet
Cell counter or hematocytometer
15‐ml conical tubes
Tabletop centrifuge
12‐well tissue culture treated plates
Filtered pipet tips
Microcentrifuge tubes
Microcentrifuge
Sterile forceps (autoclaved)
Plate reader capable of measuring absorbance

#### Co‐culture set up, infection, and supernatant collection

1 The day before infection, rinse the apical surface of each HAE culture by pipetting 250 µl PBS dropwise onto the air‐contacting, apical surface of each culture. Place into the incubator at 5% CO_2_ and 37°C for 20 min.
  This step removes accumulated mucus, which can interfere with viral infection.
2 Pre‐warm UNC ALI medium in a water bath set to 37°C.
  a. If using frozen PBMCs, rapidly thaw frozen vial in the 37°C water bath. Working in a biosafety cabinet, resuspend in warmed complete MEM. Take an aliquot of cells, count using a cell counter or hematocytometer, and pellet in a 15‐ml conical tube in a tabletop centrifuge for 5 min at 400 × *g*, room temperature.
  b. If using fresh PBMCs, working in a biosafety cabinet, take an aliquot of cells, count using a cell counter or hematocytometer, and pellet in a tabletop centrifuge in a 15‐ml conical tube for 5 min at 400 × *g*, room temperature.
3 Following counting and pelleting of fresh or frozen PBMCs, resuspend cells at 1 × 10^7^ cells/ml in warmed virus diluent. Keep cells resuspended in the 15‐ml tube until you are ready to infect.
4 Retrieve a plate containing mature HAE grown at ALI on 12‐mm Transwells from your tissue culture incubator (5% CO_2_, 37°C) and place in a clean biosafety cabinet.
  a. Ensure that you have enough 12‐mm Transwells for the conditions outlined in Fig. 2B, namely HAE‐only and HAE+PBMC co‐culture conditions, but keep in a separate plate for ease of manipulation during the infection.
  b. Rinse the apical surface of each HAE culture by pipetting 250 µl PBS dropwise onto the air‐contacting apical surface of each culture. Place into the incubator at 5% CO_2_ and 37°C for 20 min.
    This step removes accumulated mucus, which can interfere with viral infection.
5 During the incubation, prepare the viral inoculum in using the formula outlined in Basic Protocol 1, step 10.
  a. For the HAE, infection will be performed in a PBS on the air‐contacting apical surface of the culture, and the number of cells in a HAE culture in a 12‐mm Transwell is ∼1 × 10^6^ cells, which can be used to calculate viral inoculum. Plan to infect the HAE at MOI 0.5 with a total inoculum volume of 100 µl placed on the apical ALI surface.
  b. For the PBMCs, the infection will be performed in virus diluent at an MOI of 0.5 For HAE+PBMC co‐culture conditions, plan to infect a well of HAE and a well of PBMC using the corresponding medium, which will maintain an MOI of 0.5.
    PBS is used as a virus diluent for the HAE to avoid any complications of exposing the ALI to sugars and nutrients it is not normally exposed to.
6 Following the incubation in step 3, remove the PBS from the ALI of the HAE.
7 Set up infections and mock infections in HAE.
  a. Mock‐infect HAE by placing 100 µl PBS on the apical ALI surface of mock infected HAE‐only wells and co‐culture HAE wells (see Fig. 2B).
  b. Infect HAE by placing 100 µl of the viral inoculum generated in step 5 on the apical ALI surface of infected HAE‐only and co‐culture HAE wells (see Fig. 2B).
8 Set up infections and mock infections in PBMC.
  a. Mock‐infect PBMC by taking 100 µl of PBMCs resuspended in virus diluent (see step 2), mixing with 100 µl of virus diluent, and placing in a well in a 12‐well tissue culture plate. Do this for each mock‐infected PBMC‐only and HAE+PBMC co‐culture condition (see Fig. 2B).
  b. Infect PBMC by taking 100 µl of PBMCs resuspended in virus diluent (see step 2), mixing these cells with viral inoculum, and placing in a well in a 12‐well tissue culture plate. Do this for each infected PBMC‐only and HAE+PBMC co‐culture condition.
9 Take plate with infected and mock‐infected HAE and the plate with infected and mock‐infected PBMC and place both plates in a tissue culture incubator at 37°C and 5% CO_2_. Incubate for 1 hr and shake both plates every 15 min to facilitate viral infection.
10 Following this incubation, remove both plates from the incubator and place in biosafety cabinet. Proceed with the setup of the HAE+PBMC co‐culture and PBMC‐only conditions.
  a. Transfer each PBMC condition to a corresponding, labeled, sterile microcentrifuge tube, transfer to a microcentrifuge, and pellet PBMCs for 5 min at 400 × *g*, room temperature.
  b. Working in the biosafety cabinet, remove the supernatant from each tube and resuspend each pellet of PBMCs in 1 ml warmed UNC ALI medium.
  c. Transfer PBMCs to a fresh 12‐well plate by placing each condition in an individual well, according to the plate layout in Fig. 2B.
11 Remove the PBS or the viral inoculum from the ALI surface of each HAE culture.
  a. Finish setting up the HAE+PBMC co‐culture and HAE‐only conditions.
  b. Using sterile forceps, move individual HAE culture Transwell inserts to the 12‐well plate containing PBMCs.
  c. Place the culture inserts designated for HAE‐only conditions in wells containing nothing, according to the plate diagram in Fig. 2B, and then add 1 ml UNC ALI medium into the well below the insert with a sterile pipet tip.
  d. Place the HAE wells designated for co‐culture into wells containing PBMCs in UNC ALI medium designated for co‐culture, according to the plate diagram in Fig. 2B.
12 If testing the effect of the IL‐1Ra or another drug, add the compounds to the culture wells.
  a. For treated conditions, add 1 µl of IL‐1Ra (1 µg/µl) into the culture medium for each well.
  b. For vehicle treated conditions, add 1 µl of IL‐1Ra resuspension buffer into the culture medium for each well.
  c. Use sterile pipet tips and change tips with each addition.
  d. Gently shake the plate to distribute the compound or vehicle.
  e. Other compounds or concentrations of IL‐1Ra can be tested in this fashion.
    Alternatively, at the researcher's discretion, drugs or vehicle solutions can be added to UNC ALI medium prior to the HAE/PBMC‐only and HAE+PBMC co‐culture setup in steps 9 to 10, mixed well, and then dispensed to the appropriate wells during the setup of HAE/PBMC‐only and HAE+PBMC co‐culture setup in steps 9 to 10.
13 Transfer the fully setup plate following steps 10 or 11, if using IL‐1Ra or other compounds, to a tissue culture incubator at 37°C and 5% CO_2_. Incubate for 24 hr.
14 After 24 hr, remove the plate from the incubator and place in a biosafety cabinet. Add 250 µl PBS dropwise onto the air‐contacting surface of each culture to collect the apical supernatant from HAE cultures in the HAE‐only and HAE+PBMC co‐culture conditions. Place plate back into the tissue culture incubator at 37°C and 5% CO_2_ for 20 min to allow molecules present in the apical ALI to fully diffuse into the PBS.
15 Following this 20‐min incubation, remove plate from the incubator and return to the biosafety cabinet. Collect the PBS from the apical ALI surface of HAE cultures using a pipet tip and place in individual microcentrifuge tubes. This is the apical supernatant from the HAE and HAE+PBMC conditions.
16 Collect the UNC ALI medium from each well and place in individual microcentrifuge tubes. This is the basolateral supernatant from the HAE and HAE+PBMC conditions. This is the only supernatant from the PBMC‐only conditions.
17 Take all supernatants collected in microcentrifuge tubes and transfer to a microcentrifuge. Spin to remove debris and cells for 5 min at 400 × *g*, room temperature.
18 Remove the microcentrifuge tubes from the microcentrifuge. Working in a biosafety cabinet, transfer the supernatants to fresh microcentrifuge tubes.
  a. For ease of analysis, apical and basolateral supernatants from HAE‐only and HAE+PBMC co‐culture conditions in a single well can be pooled at this step, so there is one supernatant sample per condition. If pooling, adjust the volume of the PBMC‐only samples by adding 250 µl PBS, making it the same volume as the co‐culture and HAE‐only samples for equitable comparisons.
  b. If directionality of cytokine secretion is of interest (i.e., whether cytokines are released from the apical or basolateral side of the HAE culture), then keep the supernatants separate for further analysis and bring the apical supernatants to the same volume as the basolateral supernatants by adding 750 µl PBS to bring the apical supernatant volume to 1 ml for comparison.
19 Supernatants can be frozen at –20°C until ready for further analysis by ELISA.
20 When ready to perform ELISA, thaw supernatants and analyze for IL‐1β release using the Human IL‐1β ELISA Set II according to the manufacturer's protocol and for IL‐6 release using the ELISA MAX Deluxe Set Human IL‐6 according to the manufacturer's protocol. Read the results on a plate reader.
  Because these supernatants contain live SARS‐CoV‐2, the ELISAs must be performed in the biocontainment level appropriate for SARS‐CoV‐2 at your institution.

## Support Protocol 3 ESTABLISHMENT AND MATURATION OF HAE GROWN AT AN AIR–LIQUID INTERFACE

Growth of primary HBE cells at an ALI enables the establishment of organotypic primary HAE for the study of large airway epithelial cell function in vitro, including the study of the response to respiratory pathogens in these cultures. There are many different protocols for the establishment and maturation of these cultures, and we encourage the reader to investigate previously described protocols and foundational papers to gain a thorough understanding of this model system (Fulcher & Randell, 2013; Gagliardi et al., 2022; Lee et al., 2023; Randell et al., 2011). Here, we present a protocol for the establishment and maturation HAE based on the methods developed by Dr. Scott Randell and his team at UNC Chapel Hill. The use of low endotoxin, non‐proprietary UNC ALI medium (see recipe in Reagents and Solutions) in these protocols is especially critical, as this medium is essential for the success of Basic Protocol 2, which uses HAE in co‐culture with PBMCs. Alternative proprietary media may contain activators or inhibitors of cellular signal transduction pathways and may be highly stimulatory towards PBMCs even at baseline in the absence of SARS‐CoV‐2 infection (also see below in the Commentary section). For these cultures, plastics including tissue culture dishes and permeable Transwell membrane supports are coated with collagen, then primary HBE cells are thawed and grown in a dish to expand cell number and then passaged HBE cells are seeded onto Transwell supports. After reaching confluency, an ALI interface is established, and cultures are fed and matured until they reach 28 to 35 days of age. At this point, the HAE cultures will be ready for the experiment described in Basic Protocol 2. Please note that some of the reagents below can also be purchased directly from the UNC Marsico Lung Institute Tissue Procurement and Cell Culture Core (https://www.med.unc.edu/marsicolunginstitute/core‐facilities/tissueprocurmentandcellculturecore/) in addition to the commercial sources listed. This is indicated in the materials list below.

### Materials

PureCol solution (Advanced Biomatrix, cat. no. 5005)
Human collagen type IV solution (see recipe)
H_2_O, sterile (Gibco, cat. no. 15230001)
Bronchial epithelial growth medium (BEGM) (Lonza, cat. no. CC3170 or UNC MLI Tissue Culture and Procurement Core)
Early passage primary HBE cells (e.g., Lonza, cat. no. CC2540S, UNC MLI Tissue Culture and Procurement Core, or equivalent)
70% ethanol in water (Fisher, cat. no. BP82011)
Trypan blue
PBS (e.g., Gibco, cat. no. 10010023)
Accutase (Stem Cell Technologies, cat. no. 07920)
UNC ALI medium (see recipe)

10‐cm tissue culture treated dishes
12‐mm Transwell culture inserts (Corning, cat. no. 3460)
Type II class A2 biosafety cabinet with UV light
Incubator, 37°C, 5% CO_2_
Aspirator
Sterile aspirator pipettes
4°C storage
Water bath, 37°C
15‐ml conical tubes
Centrifuge
Cell counter or hematocytometer
Inverted microscope
12‐well tissue culture treated plates
Sterile forceps

#### Collagen coating cell growth surfaces

1 At least 24 hr prior to thawing primary HBE, plan to coat 10‐cm tissue culture dishes and 12‐mm Transwell membrane supports with PureCol and human collagen type IV solution, respectively.
2 For tissue culture plates:
  a. Dilute PureCol 1:75 in sterile water, working in a biosafety cabinet.
  b. Add 3 ml to each 10‐cm tissue culture dish you desire to coat.
  c. Place in a tissue culture incubator at 37°C and 5% CO_2_ for 2 to 24 hr.
  d. After this timeframe, remove any additional liquid with an aspirator in the biosafety cabinet, allow to dry in the biosafety cabinet with the blower on, and then expose the plates to UV light for at least 30 min with the sash closed in the biosafety cabinet.
  e. Package plates for storage at 4°C for up to 8 weeks.
3 For 12‐mm Transwell permeable membrane supports:
  a. Add 150 µl of human collagen type IV solution onto the apical surface of each Transwell support, working in a biosafety cabinet.
  b. Dry overnight in the biosafety cabinet with the blower on.
  c. The next day, expose to UV light for 30 min with the sash of the biosafety cabinet closed.
  d. Open the cabinet with the blower on.
  e. Package the Transwell supports for storage at 4°C for up to 6 weeks.

#### Thawing and subculture of primary HBEs

4 Warm an aliquot of BEGM, ∼20 ml for 1 vial of 0.5–2 × 10^6^ cells, in a water bath at 37°C.
5 Thaw cryopreserved vial of primary HBE rapidly in a water bath at 37°C. Remove the vial and take to a biosafety cabinet just before the sample melts fully.
6 Spray the outside of the cryovial with 70% ethanol to decontaminate. Working in a biosafety cabinet, place thawed HBE cell suspension into a 15‐ml conical tube and then slowly add 9 ml of warmed BEGM to maximize cell viability. Take a small aliquot of cells for counting.
7 Centrifuge for 5 min at 400 × *g*, room temperature, to remove cryopreservation medium.
8 Working in a biosafety cabinet, remove the supernatant and resuspend the cell pellet a concentration of ∼1 × 10^6^ cells/ml, based on the number of cells frozen.
9 Reserve a small aliquot of the resuspended cells. Then, count the reserved cells diluted 50:50 in trypan blue to determine the total viable cell number after thawing.
10 After obtaining the count, plan to plate ∼1 × 10^6^ cell per 10‐cm dish. If you have fewer cells, plate these in one 10‐cm dish. If you have significantly more cells, divide into two or more 10‐cm dishes as needed. Bring the cell suspension up to 10 ml of total volume per dish to be plated (10 ml for 1 plate, 20 ml for 2 plates, etc.).
11 Place the cell suspension into a PureCol‐coated 10‐cm dish and gently shake to evenly distribute the cells (up/down and left/right). Place the dish into a tissue culture incubator at 37°C and 5% CO_2_ overnight. Label the dish with the cell identity, seeding date, passage number, and number of viable cells seeded.
  If cells have not been cultured previously (i.e., directly isolated from the tissue donor), these cells are considered at passage 0 (P0). Otherwise, use the passage number indicated on the cryovial. This protocol works best with cells that are P1 or P2.
12 Change the medium after 24 hr by aspirating the old BEGM and adding 10 ml fresh BEGM.
13 Monitor by inverted microscope daily for health and confluency. Change the medium every other day until the cells are 70% to 90% confluent. This may take up to 7 days.
14 When the HBE are confluent, they can either be lifted for plating into Transwells for ALI culture preparation or expanded further. If your cells are at P1 or P2, you can move forward to set up ALI cultures.
  If desired, P0 and P1 cells can be cryopreserved for future use.
15 To lift HBE cells, begin by removing culture medium, placing 5 ml PBS in the culture dish to rinse, and then removing the PBS, while working in a biosafety cabinet. Following this, add 3 ml Accutase to the dish and place in a tissue culture incubator at 37°C and 5% CO_2_ to detach cells.
16 Incubate for 5 min, tap the dish gently to release the cells, and then assess whether the cells are detached under a tissue culture microscope. Incubate further if cells are not detached.
17 Once cells have released, transfer the cell suspension to a 15‐ml conical tube and centrifuge 5 min at 400 × *g*, room temperature.
18 After centrifugation, working in a biosafety cabinet, remove the supernatant and resuspend the cell pellet a concentration of ∼1 × 10^6^ cells/ml.
19 Reserve a small aliquot of the resuspended cells. Then, count the reserved cells diluted 50:50 in trypan blue to determine the total viable cell number.
20 If passaging further in 2D culture, resuspend the cells in 10 ml BEGM and plate in a new PureCol‐coated 10‐cm dish and return to step 11 above for an additional passage. Otherwise, skip this step and proceed to step 21.
21 If preparing to seed 12 mm Transwells for ALI HAE cultures, plan to seed 1.25–2.50 × 10^5^ cells per Transwell in a volume of 500 µl delivered to the apical surface of the Transwell. Thus, resuspend the cell pellet in the appropriate amount of medium for the number of cells grown and desired number of HAE cultures.
  For instance, a total cell number of 3 × 10^6^ cells could be resuspended in 6 ml medium to seed 12 Transwell supports at a density of 2.50 × 10^5^ cells per culture for 12 mature HAE cultures or the same number of cells could be resuspended in 12 ml to seed 24 Transwell supports at a density of 1.25 × 10^5^ cells per culture for 24 mature HAE cultures. Adjust according to your needs and number of cells grown.
22 Working in a biosafety cabinet, place coated Transwell supports into a 12‐well tissue culture plate using sterile forceps. If you need to put the forceps down or re‐sterilize during use, place in a 50 ml conical tube filled with 70% ethanol and shake to dry by evaporation after sanitizing.
23 After placing the desired number of Transwell supports into the culture plates, place 500 µl of the HBE cell suspension into the apical surface of each Transwell. Pipet the mixture up and down prior to and during the plating process to ensure that all Transwell supports receive the same number of cells in UNC ALI medium.
24 After placing the cell suspension on the apical surface of the Transwell supports, inspect the well below the Transwell for any drops of cell suspension that may have dripped. Use an aspirator to remove any cell suspension in the wells below the Transwell as needed.
25 Add 1 ml UNC ALI medium to the well below the Transwell support, referred to as the basolateral compartment.
26 Place the plate in a tissue culture incubator at 37°C and 5% CO_2_ overnight.
27 The next day change the medium in the apical and basolateral compartments. Working in a biosafety cabinet, aspirate old medium from both compartments. Rinse the apical surface with a few drops of PBS (100 to 200 µl), aspirate the PBS, and place a few drops of UNC ALI medium (100 to 200 µl) on the apical surface. Then, add 1 ml fresh UNC ALI medium to the basolateral compartment.
  Change tips between the apical and basolateral surfaces to prevent growth of cells in the well instead of on the Transwell support.
  Lower medium volume in the apical surface is necessary to support differentiation. Do not fill the Transwell apical compartment with medium.
28 Inspect the cells daily to assess confluence. This usually happens ≤7 days after seeding the Transwell supports. Change the apical and basolateral medium as directed in step 26 every other day until cells are confluent.
29 Once the cultures are confluent, remove medium from the apical compartment, bringing the cultures to an ALI. Change the basolateral medium.
30 Continue changing the basolateral medium every other day and rinse the apical surface with PBS once a week. Perform this rinse by adding a few drops of PBS and then aspirating the PBS away, then proceed with a basolateral medium change.
  During the differentiation process, HAE cultures will begin to make mucus, which is visible at the apical ALI. PBS rinses prevent excess mucus accumulation. The amount of mucus varies between donors.
31 After 28 days in culture, these HAE ALI cultures are ready for infection. SARS‐CoV‐2 efficiently infects these cultures between 28 and 35 days in culture (beginning after step 23) and performing Basic Protocol 2 with these cultures is recommended during this timeframe.
  Well‐differentiated HAE ALI cultures will have mature ciliated epithelial cells that can be observed by light microscopy. It is easier to see cilia beating after PBS washing the apical surface.

## REAGENTS AND SOLUTIONS

### Complete MEM with 10% FBS

Prepare a 500‐ml bottle of MEM (Gibco, cat. no. 11095080) containing:
10% (v/v) FBS (50 ml of 100% stock, e.g., Corning, cat. no. 35016CV)
1 mM sodium pyruvate (5 ml from 100 mM stock, Gibco, cat. no. 11360070)
1% (v/v) GlutaMAX Supplement (5 ml of 100% stock, Gibco, cat. no. 35050061)
1% (v/v) penicillin‐streptomycin (5 ml of 100% stock, Gibco, cat. no. 15070063)
1% (v/v) MEM non‐essential amino acids solution (5 ml of 100% stock, Gibco, cat. no. 11140035)
Mix well in a biosafety cabinet to maintain sterility
Store up to 2 months at 4°C

### Freezing medium

90% FBS (e.g., Corning, cat. no. 35016CV)
10% DMSO (Sigma, cat. no. D2650‐100ML)
Mix well in a biosafety cabinet to maintain sterility
Store up to 1 month at 4°C

### Human IL‐1Ra, 1 µg/µl

100 µg human IL‐1RA recombinant protein (PeproTech, cat. no. 200‐01RA‐100UG)
100 µl IL‐1Ra resuspension buffer (see recipe)
Mix well and sterilize with a 0.2‐µm filter in a biosafety cabinet
Make 10‐µl aliquots
Store up to 6 months at –80°C
Do not freeze/thaw
Use at a final concentration of 1 µg/ml

### Human type IV collagen solution

10 mg human type IV collagen (Sigma, cat. no. C‐7521)
20 ml double distilled water to make a 0.5 mg/ml solution
50 µl glacial acetic acid (Sigma, cat. no. A6283‐100ML)
Dissolve for 4 to 8 hr at 37°C
Sterile filter in a biosafety cabinet using a 0.2‐µm syringe filter
This creates a 10× stock
Make 1‐ml aliquots
Store up to 6 months at –20°C
Do not freeze/thaw
Thaw and dilute stock 1:10 in sterile water (Gibco, cat. no. 15230001) for a final concentration of 0.05 mg/ml

### IL‐1Ra resuspension buffer

0.1% BSA (w/v) (Sigma, cat. no. A2058‐5G) in PBS (e.g., Gibco, cat. no. 10010023)
Filter sterilize using 0.2‐µm vacuum bottle filter (Thermo Scientific, cat. no. 5670020)
Store up to 2 months at 4°C

### Low endotoxin UNC ALI medium (adapted from Fulcher & Randell, 2013)

Working in plasticware that has not been used for other purposes, create a base medium of 500 ml of 1× LHC basal medium (Gibco, cat. no. 12677019) mixed 1:1 with 500 ml of 1× Dulbecco's modified Eagle medium (DMEM) (Corning, cat. no. 10‐013‐CV) and add:

1% (v/v) penicillin‐streptomycin (10 ml of 100% stock, Gibco, cat. no. 15070063)
0.5 mg/ml BSA (Sigma, cat. no. A2058‐100G)
10 µg/ml bovine pituitary extract (BPE) (10 ml of 1 mg/ml in PBS; Sigma, cat. no. P1167)
5 µg/ml insulin [0.5 ml of 10 mg/ml stock: mix 95 ml deionized water and 5 ml glacial acetic acid (Fisher, cat. no. A38‐212) in a beaker, add 500 mg insulin (Sigma, cat. no. I0516), aliquot, and store at –20°C]
10 µg/ml transferrin (1 ml of 10 mg/ml stock in PBS stored at –20°C, Sigma, cat. no. T0665)
0.1 µM hydrocortisone (1 ml of 0.1 mM stock in deionized water, Sigma, cat. no. H0396)
0.01 µM triiodothyronine [1 ml of 0.01 mM stock: dissolve 6.3 mg triiodothyronine (Sigma, cat. no. T6397) in 5 ml of 0.2 M NaOH, bring volume to 1 L with deionized water, aliquot, and store at –20°C]
0.6 µg/ml epinephrine [1 ml of 0.6 mg/ml stock: dissolve 50 mg room temperature epinephrine (Sigma, cat. no. E4642) in 10 ml of 0.1 N HCl, bring volume to 100 ml with deionized water, aliquot, and store at –20°C]
0.5 ng/ml epidermal growth factor (EGF) (20 µl of 25 µg/ml stock in PBS stored at –20°C, Gibco, cat. no. PHG0313)
50 nM retinoic acid [make a concentrated 1 mM stock in ethanol, dilute to a 1000× stock in 1% (v/v) BSA in PBS, and then add 1 ml to the medium mixture, Sigma, cat. no. R2625]
0.5 µM phosphorylethanolamine (1 ml of 0.5 mM stock in PBS stored at –20°C, Sigma, cat. no. P0503)
0.5 µM ethanolamine [1 ml of 0.5 mM stock in PBS: 6 µl ethanolamine (Sigma, cat. no. E0135) in 200 ml PBS, stored at –20°C]
3 µM zinc sulfate (1 ml of 3 mM stock in deionized water, Sigma, cat. no. Z0251)
0.1% (v/v) metals stock (see recipe)
0.1% (v/v) trace elements stock (see recipe)
Filter sterilize with 0.2‐µm vacuum bottle filter (Thermo Scientific, cat. no. 5670020) in a biosafety cabinet
Store up to 2 months at 4°C
  Please note that low endotoxin UNC ALI medium can also be purchased from the UNC Marsico Lung Institute Tissue Procurement and Cell Culture Core at UNC Chapel Hill (https://www.med.unc.edu/marsicolunginstitute/core‐facilities/tissueprocurmentandcellculturecore/).
  Working with fresh, disposable plasticware minimizes potential endotoxin contamination.
  Do not use glass vessels, stir bars, or any other potential source of endotoxin contamination.
*Please note that endotoxin‐free BSA is used*.
  Keep retinoic acid protected from light by wrapping containers in foil.

### Metals stock, 1000× (adapted from Fulcher & Randell, 2013)

Combine in 800 ml distilled water:
0.42 g FeSO_4_·7H_2_O (Sigma, cat. no. F8048) [Final] = 15 µM
122.0 g MgCl_2_·6H_2_O (Avantor, cat. no. JT2444‐1) [Final] = 600 µM
16.17 g CaCl_2_·2H_2_O (Sigma, cat. no. C3881) [Final] = 110 µM
5 ml HCl (12 N, Sigma, cat. no. 320331)
Stir and bring the volume to 1 L with distilled water
Filter sterilize using 0.2‐µm vacuum bottle filter (Thermo Scientific, cat. no. 5670020) in a biosafety cabinet
Aliquot and store at −20°C for up to 1 year

### Trace elements stock (adapted from Fulcher & Randell, 2013)

Make 100 ml stock solutions of all trace elements in deionized water listed below:
520 mg selenium (NaSeO_3_, Sigma, cat. no. S5261) [Stock] = 300 mM, [Final] = 30 mM
20 mg manganese (MnCl_2_·4H_2_O, Sigma, cat. no. M5005) [Stock] = 10 mM, [Final] = 1 mM
14.2 g silicone (Na_2_SiO_3_·9H_2_O, Sigma, cat. no. S5904) [Stock] = 5 M, [Final] = 500 mM
124 mg molybdenum ([(NH_4_)_6_Mo_7_O_24_·4H_2_O], Sigma, cat. no. M1019) [Stock] = 10 mM, [Final] = 1 mM
59 mg vanadium (NH_4_VO_3_, Sigma, cat. no. 398128) [Stock] = 50 mM, [Final] = 5 mM
26 mg nickel (NiSO_4_·6H_2_O, Sigma, cat. no. N4882) [Stock] = 10 mM, [Final] = 1 mM
11 mg tin (SnCl_2_·2H_2_O, Sigma, cat. no. S9262) [Stock] = 5 mM, [Final] = 500 µM
After making stock solutions of each trace element, fill a flask with 992 ml deionized water
Add 1 ml of each trace element stock
Add 1 ml HCl (12 N, Sigma, cat. no. 320331)
Filter sterilize using 0.2‐µm vacuum bottle filter (Thermo Scientific, cat. no. 5670020) in a biosafety cabinet
Store up to 1 year at room temperature

### Virus diluent with 2% FBS

Prepare a 500‐ml bottle of MEM (Gibco, cat. no. 11095080) containing:
2% (v/v) FBS (50 ml of 100% stock, e.g., Corning, cat. no. 35016CV)
1 mM sodium pyruvate (5 ml of 100 mM stock, Gibco, cat. no. 11360070)
1% (v/v) GlutaMAX supplement (5 ml of 100% stock, Gibco, cat. no. 35050061)
1% (v/v) penicillin‐streptomycin (5 ml of 100% stock, Gibco, cat. no. 15070063)
1% (v/v) MEM non‐essential amino acids solution (5 ml of 100% stock, Gibco, cat. no. 11140035)
Mix well in a biosafety cabinet to maintain sterility
Store up to 2 months at 4°C

### VX‐765 in solution, 20 mg/ml, 1000×

10 mg VX‐765 (Invivogen, cat. no. inh‐vx765i‐1)
5 ml DMSO (Sigma, cat. no. D2650‐100ML)
Mix well in a biosafety cabinet to maintain sterility
Make 200‐µl aliquots
Store at −20°C for up to 6 months
Do not freeze/thaw aliquots
Use at final concentration of 20 µg/ml

## COMMENTARY

### Background Information

Interactions between epithelial and immune cell populations in the lung during SARS‐CoV‐2 infection are critical for stimulating inflammatory responses to viral infection and give rise to immune responses not observed in individual cell types. While animal models provide some avenue for studying intercellular communication, in vitro cell culture models offer several, distinct advantages that may better serve certain lines of interrogation. First, in vitro models allow for work with primary human cells, which have higher fidelity to the mechanisms of human biology in SARS‐CoV‐2 infection. Second, these models allow for higher‐throughput and lower‐cost experiments compared to animal models and are better suited for larger‐scale experiments including drug screens and gene‐deletion screens. Furthermore, in vitro models permit mechanistic inquiries into molecule trafficking between cell types and the ability to study specific cell–cell interactions in isolation. Because of these advantages, we developed these in vitro models to study epithelial‐immune interactions in SARS‐CoV‐2 infection, which we detail in this article.

In Basic Protocol 1, we describe a partially primary co‐culture system of Vero‐E6 cells, which an epithelial cell line that supports SARS‐CoV‐2 infection, and PBMCs, which contain many inflammatory immune cell populations including monocytes and dendritic cells, to study epithelial‐immune interactions during SARS‐CoV‐2 infection. This culture system has higher flexibility in terms of timing and cost compared to the fully primary co‐culture system outlined in Basic Protocol 2, and it enables study of how immune cells response to infected epithelial cells in culture. However, this system is not ideal to study how epithelial cells respond to signals from immune cells, as Vero‐E6 cells do not have high fidelity to primary human tissues, which was a motivation for developing the fully primary human co‐culture system outlined in Basic Protocol 2. To enable the reader to fully execute Basic Protocol 1, we provide Support Protocols 1 and 2, which outline how to culture Vero‐E6 cells and how to isolate and store PBMCS, respectively.

In Basic Protocol 2, we detail a fully primary, human co‐culture system of organotypic, bronchial HAE with PBMCs as an additional model to study epithelial‐immune interactions during SARS‐CoV‐2 infection. This culture system offers higher fidelity to humans and enables the study of how the infected airway epithelium responds to cues from the immune compartment. However, given the long differentiation time of HAE and the costs associated with primary human cell culture, some researchers may opt for the simpler, cost‐effective system outlined in Basic Protocol 1 instead. To support execution of Basic Protocol 2, we provide Support Protocol 3, which describes how to establish and differentiate primary HAE cultures, and highly encourage those interested in the system to read additional descriptions of this widely used primary HAE culture system (Fulcher & Randell, 2013; Gagliardi et al., 2022; Lee et al., 2023; Randell et al., 2011).

### Critical Parameters

There are several critical parameters relevant to both or either Basic Protocols 1 and 2. One critical parameter for both Basic Protocols 1 and 2 is the timing of these experiments, which we describe fully in the Time Considerations section below, as coordinating culture and infection of multiple cell types is essential for the success of these protocols. Second, donor variability, particularly regarding differences in PBMC donors, is relevant to both Basic Protocols 1 and 2. This is likely due to both genetic differences between the PBMC donors and differences in relative amounts of different cell types contained within a PBMC prep from a given blood draw. We suggest performing these experiments with several different PBMC donors to get an understanding of the range of the magnitude cytokine responses measured in these systems. Third, the presence of bacterial contamination, including the presence of mycoplasma, can activate IL‐1β signaling and will obscure the results in Basic Protocols 1 and 2. Thus, the user should routinely monitor cultured cells for contamination, including mycoplasma.

Regarding Basic Protocol 1, specific considerations must be made regarding the health and culture age of the Vero‐E6 cells in addition to the considerations above. In general, early passage number (<30 passages) Vero‐E6 behave more predictably in this co‐culture system than later passage cells, as higher passage Vero‐E6 cells may show higher baseline levels of IL‐1β in co‐culture with PBMCs in the absence of viral infection. Similarly, Vero‐E6 cells that have been in continuous culture for >6 weeks may also show higher baseline levels of IL‐1β in co‐culture with PBMCs in the absence of viral infection. Thus, we recommend using lower passage Vero‐E6 cells that are in culture for <6 weeks to ensure the success of Basic Protocol 1.

In addition to the considerations outlined in the first paragraph of this section, medium composition is essential to the success of Basic Protocol 2. While it is standard for the MEM used in Basic Protocol 1 to be free of endotoxin and other factors that can activate spontaneous cytokine signaling in immune cells, this is not consistently true for media tailored to airway epithelial cells, which lack the machinery to respond to lipopolysaccharide (LPS), a major component of endotoxin (Jia et al., 2004). Commercially available media for ALI culture of HBEs may lead to spontaneous activation of IL‐1β and IL‐6 signaling from PBMCs due to a lack of screening for endotoxin or due to the inclusion of other signaling molecules. Please note that commercial HBE ALI medium may not include a description of its composition, which may be considered proprietary knowledge by the company. This makes it very difficult to determine if there are factors that will interfere in the co‐culture system without direct testing. If you will use medium other than what is listed in Basic Protocol 2, please confirm that medium used is endotoxin‐free and pre‐test medium used in Basic Protocol 2 to determine if PBMCs will spontaneously produce IL‐1β/6 after incubation in medium for the duration of Basic Protocol 2 (24 hr) prior to performing the full protocol. Please note that the composition of UNC ALI medium described in the Reagents and Solutions section is low‐endotoxin and uses components screened for the presence of endotoxin, which is why this is our medium of choice for Basic Protocol 2. Using different components other than those listed may lead to the inclusion of endotoxin. Testing of medium for spontaneous PBMC IL‐1β/IL‐6 responses by incubating in the medium for the duration of Basic Protocol 2 (24 hr) prior to performing the full protocol is recommended with each batch, especially if deviations from the listed catalog numbers are used.

### Troubleshooting

In Table 1, we outline common problems found in Basic Protocols 1 and 2 along with possible causes and solutions.

**Table 1:** Troubleshooting Guide for Co‐Culture Systems in Basic Protocols 1 and 2

Problem	Possible Cause	Solution
Low or no IL‐1β/IL‐6 responses with SARS‐CoV‐2 infection in co‐culture	PBMC donor variation	Check responses from several PBMC donors
	High Vero‐E6 age in culture (Basic Protocol 1)	Thaw new Vero‐E6 cells at a low passage number
		Do not keep Vero‐E6 cells in culture for over 6 weeks
	Lack of CPE in Vero‐E6 cells (Basic Protocol 1)	Test multiple time points if you do not observe CPE in Vero‐E6 cells at 72/96 HPI
	High levels of mucus interfering with SARS‐CoV‐2 infection in HAE (Basic Protocol 2)	Ensure that mucus has been thoroughly removed from the ALI surface by PBS washing
	Issues with SARS‐CoV‐2 viral stocks	Check viral titer and infectivity of stock by plaque assay (Mendoza et al., 2020) or similar method
Spontaneously high IL‐1β/IL‐6 responses in the absence of SARS‐CoV‐2 infection	Contamination	Check all cells in the co‐culture system for contamination, including mycoplasma; discard all affected cells, medium, and associated culture reagents
	Endotoxin or pro‐inflammatory signals in medium	Test if medium alone stimulates IL‐1β/IL‐6 responses in culture prior to use in co‐culture system and discard if stimulatory; only prepare medium with low‐endotoxin reagents and test all commercially sourced products

### Understanding Results

With Basic Protocols 1 and 2, one can expect to observe cytokine responses dependent on viral infection and co‐culture of epithelial and immune cell types by ELISA. In Basic Protocol 1, co‐culture of Vero‐E6 with PBMCs during SARS‐CoV‐2 infection will yield 10 to 500 pg/ml IL‐1β, as measured by ELISA, where the magnitude of this response varies between PBMC donors. Notably, the inclusion of inhibitors in these infected co‐culture conditions, such as addition of 20 µg/ml VX‐765, may block IL‐1β release in this context. This would be expected with VX‐765 inclusion, because it inhibits caspase‐1 activity necessary for IL‐1β release (Wannamaker et al., 2007). IL‐1β release is not expected to occur in conditions that lack viral infection or conditions that lack both cell types, as our previous work demonstrated that epithelial‐immune interactions during infection were critical for robust IL‐1β responses (Barnett et al., 2023). In Basic Protocol 2, co‐culture of HAE ALI cultures with PBMCs during SARS‐CoV‐2 infection will stimulate both IL‐1β and IL‐6 responses, as measured by ELISA, but not in conditions lacking both cell types or in conditions lacking viral infections. One can expect that IL‐1β responses as measured by ELISA will vary due to differences in PBMC donors, but we find that the same donors may show slightly lower values (10 to 100 pg/ml IL‐1β) (Barnett et al., 2023). Similarly, IL‐6 responses in this system will vary between donors but range between 50 and 2000 pg/ml, as measured by ELISA. Inclusion of the IL‐1Ra in this system will block IL‐1‐dependent IL‐6 responses in conditions with both cell types and SARS‐CoV‐2 infection. While here we describe the inclusion of compounds known to block these cytokine responses, VX‐765‐mediated inhibition of IL‐1β release in Basic Protocol 1 and IL‐1Ra‐mediated inhibition of IL‐6 release in Basic Protocol 2, one can assess the efficacy of other compounds of altering these responses in a similar fashion and interpret loss of cytokine release upon SARS‐CoV‐2 infection in co‐culture conditions to mean that the drug potentially inhibits these responses.

### Time Considerations

Timelines are provided for Basic Protocols 1 and 2 to assist with planning and understanding of the time requirements necessary to execute these experiments. However, one must also account for the cell culture work prior to execution of these Basic Protocols 1 and 2. Here, we will describe the considerations for each of these Basic Protocols and along with their associated support protocols. Please note that differences in institutional biosafety procedures for SARS‐CoV‐2 handling may influence these time estimates.

Basic Protocol 1 will run for 120 hr (5 days), as shown in the timeline (Fig. 1A). For this, Vero‐E6 cells are plated ∼24 hr prior to the start of the experiment, plated Vero‐E6 cells are then infected and incubated for 72 hr, and then Vero‐E6 cells are co‐cultured with or without PBMCs for another 24 hr (total of 96 hr in infection). For this first phase, plating of Vero‐E6 cells will take ∼1 hr. For the following day, infection of Vero‐E6 cells will take ∼2 hr including set up and takedown of the infection process and the 1‐hr infection. At the 72‐hr co‐culture timepoint, the user can expect to spend ∼3 hr thawing PBMCs, performing an infection in PBMCs, and setting up the co‐culture conditions. At the 96‐hr final timepoint, the user will spend ∼1 hr harvesting culture supernatants. In addition to this 5‐day time course, one will need to thaw and subculture Vero‐E6 cells prior to plating, which can take up to 10 days, as outlined in Support Protocol 1. Likewise, preparation and freezing of PBMCs, described in Support Protocol 2, usually takes 3 to 4 hr, but this can be done well in advance of the experiment. Following the infection co‐culture outlined in Basic Protocol 1, the user will assess IL‐1β release by ELISA, which will require a 30‐min coating step, followed by an overnight incubation, and running the assay the next day for ∼6 hr.

Execution of Basic Protocol 2 will take ∼48 hr to complete, as indicated in the experiment timeline (Fig. 2B). Over this 48‐hr period, the timing of specific steps includes the rinsing of HAE cultures the day prior to infection to remove mucus, a step takes ∼30 min to execute, then the set up of the HAE‐PBMC co‐culture and infections the following day, a step that will take ∼4 hr to execute, and then the takedown of the experiment and collection of supernatants, which will take ∼1 hr. A key time consideration of Basic Protocol 2 is the timing of the maturation of primary HAE cultures. As outlined in Support Protocol 3, the primary HAE cultures need to mature for at least 28 days after being seeded onto permeable membrane supports, a step that occurs ∼1 week after thawing primary HBE cells and growth on a plastic plate. Notably, these cultures must be used for SARS‐CoV‐2 infection between 28 and 35 days of growth, as older cultures show less consistent responses and have higher risk of contamination. Thus, planning the experiment around when these HAE cultures are ready for infection is paramount, and the user should plan these experiments at least 6 weeks in advance to ensure all the cells will be ready. As with Basic Protocol 1, PBMCs will take 3 to 4 hr to isolate but can be stored frozen in liquid nitrogen well in advance of execution of Basic Protocol 2. After execution of the co‐culture outlined in Basic Protocol 2, cell culture supernatants can be stored at –20°C until ready for analysis by ELISA. Both the IL‐1β and IL‐6 ELISAs will take ∼6 hr to execute after performing an overnight coating step the day prior to performing the ELISAs.

### Author Contributions

**Scott Randell**: Funding acquisition; methodology; writing—review and editing. **Katherine Barnett**: Conceptualization; funding acquisition; investigation; methodology; project administration; supervision; writing—original draft; writing—review and editing.

### Conflict of Interest

Dr. Randell directs the Marsico Lung Institute Tissue Procurement and Cell Culture Core that can provide reagents used in the described protocols on a fee for service basis. All other authors have no conflicts to disclose.

## Data Availability

Data sharing is not applicable to this article as no new data were created or analyzed in this study.
